# Histone deacetylase inhibitors modulate K_ATP_ subunit transcription in HL-1 cardiomyocytes through effects on cholesterol homeostasis

**DOI:** 10.3389/fphar.2015.00168

**Published:** 2015-08-13

**Authors:** Naheed Fatima, Devin C. Cohen, Gauthaman Sukumar, Tristan M. Sissung, James F. Schooley, Mark C. Haigney, William C. Claycomb, Rachel T. Cox, Clifton L. Dalgard, Susan E. Bates, Thomas P. Flagg

**Affiliations:** ^1^Department of Anatomy, Physiology and Genetics, F. Edward Hebert School of Medicine, Uniformed Services University of the Health SciencesBethesda, MD, USA; ^2^Developmental Therapeutic Branch, National Cancer Institute, National Institutes of HealthBethesda, MD, USA; ^3^Department of Medicine, F. Edward Hebert School of Medicine, Uniformed Services University of the Health SciencesBethesda, MD, USA; ^4^Department of Biochemistry and Molecular Biology, LSU Health Sciences CenterNew Orleans, LA, USA; ^5^Department of Biochemistry, F. Edward Hebert School of Medicine, Uniformed Services University of the Health SciencesBethesda, MD, USA

**Keywords:** epigenetics, *Abcc8*, *Abcc9*, romidepsin, cholesterol, SREBP

## Abstract

Histone deacetylase inhibitors (HDIs) are under investigation for the treatment of a number of human health problems. HDIs have proven therapeutic value in refractory cases of cutaneous T-cell lymphoma. Electrocardiographic ST segment morphological changes associated with HDIs were observed during development. Because ST segment morphology is typically linked to changes in ATP sensitive potassium (K_ATP_) channel activity, we tested the hypothesis that HDIs affect cardiac K_ATP_ channel subunit expression. Two different HDIs, romidepsin and trichostatin A, caused ~20-fold increase in SUR2 (*Abcc9*) subunit mRNA expression in HL-1 cardiomyocytes. The effect was specific for the SUR2 subunit as neither compound causes a marked change in SUR1 (*Abcc8*) expression. Moreover, the effect was cell specific as neither HDI markedly altered K_ATP_ subunit expression in MIN6 pancreatic β-cells. We observe significant enrichment of the H3K9Ac histone mark specifically at the SUR2 promoter consistent with the conclusion that chromatin remodeling at this locus plays a role in increasing SUR2 gene expression. Unexpectedly, however, we also discovered that HDI-dependent depletion of cellular cholesterol is required for the observed effects on SUR2 expression. Taken together, the data in the present study demonstrate that K_ATP_ subunit expression can be epigenetically regulated in cardiomyocytes, defines a role for cholesterol homeostasis in mediating epigenetic regulation and suggests a potential molecular basis for the cardiac effects of the HDIs.

## Introduction

Histone deacetylase inhibitors (HDIs) have emerged as potential therapeutic agents for the treatment of cancer and heart disease (Ma et al., 2009; McKinsey, 2012). Histone deacetylases (HDACs) are known to be key co-factors of the machinery that regulates gene transcription. HDACs typically work in concert with DNA binding proteins to remove covalently attached acetyl groups from lysine residues contained within histone proteins, resulting in less accessible chromatin and reduced gene transcription (Kouzarides, 2007), however, this simple model of HDI action may belie the diverse effects of HDIs that can affect both normal and cancerous cell viability (Falkenberg and Johnstone, 2014).

To date, four HDIs—romidepsin, vorinostat, belinostat, and panobinostat—have been approved by the FDA for treating heamatologic malignancies including refractory cutaneous T-cell lymphoma or multiple myeloma (Mann et al., 2007; Piekarz et al., 2011; Coiffier et al., 2012; Lee et al., 2015; Richardson et al., 2015). While HDIs have proven successful, cardiac safety has been a concern. Non-specific ST segment changes, long QT, atrial fibrillation, and torsades de pointes have all been reported in clinical trials (Sandor et al., 2002; Shah et al., 2006; Stadler et al., 2006; Steele et al., 2008; Lynch et al., 2012; Noonan et al., 2013). In a recent trial with romidepsin, the most common ECG finding was ST segment morphological changes with no evidence of associated myocardial ischemia (Noonan et al., 2013). ST segment elevation and depression are typically associated with the activation of ATP-sensitive (K_ATP_) channels during ischemia (Kubota et al., 1993; Li et al., 2000), suggesting that HDIs may specifically affect K_ATP_ channel subunit expression in cardiac myocytes.

HDIs have also shown promise in the treatment of cardiovascular disease (McKinsey, 2012). Trichostatin A (TSA) interferes with phenylephrine-induced hypertrophy of neonatal ventricular myocytes (Cao et al., 2011) and studies in animal models have shown that HDIs inhibit the development of cardiac hypertrophy due to angiotensin II infusion, aortic banding or pressure overload (Kee et al., 2006; Kong et al., 2006). HDIs have also been shown to inhibit the expression of hypertrophic genes, inflammatory markers, and fibrosis in the diseased heart (Liu et al., 2008; Cardinale et al., 2010; Cao et al., 2011). Interestingly, HDIs have also been shown to reduce infarct size and maintain contractile function following ischemia-reperfusion, similar to the effects of pharmacological or ischemic preconditioning (Zhao et al., 2007; Xie et al., 2014), which again is associated with activation of sarcolemmal K_ATP_ channels (Suzuki et al., 2002).

Based on these observations, we hypothesized that HDIs may specifically affect the expression of K_ATP_ channel subunits. A single K_ATP_ channel is a hetero-octomeric protein complex comprised of four pore-forming, inward rectifier (Kir6.1 or Kir6.2) and four sulfonylurea receptor (SUR1 or SUR2) subunits that are encoded by four separate genes (Inagaki et al., 1995a,b, 1996; Chutkow et al., 1999). Specific subunit combinations make up K_ATP_ channels in different tissues. For example, K_ATP_ channels in mouse atrial cardiomyocytes and pancreatic β-cells are composed of SUR1 and Kir6.2, while SUR2A and Kir6.2 form K_ATP_ in ventricular myocytes (Inagaki et al., 1995b, 1996; Flagg et al., 2008). There are relatively few studies to identify factors that regulate K_ATP_ channel subunit expression and many of these have been focused on pancreatic β-cell models rather than cardiomyocytes (Ashfield and Ashcroft, 1998; Hernandez-Sanchez et al., 1999; Kim et al., 2002). One study focused on myocardium demonstrated an important role for forkhead transcription factors in regulating K_ATP_ expression (Philip-Couderc et al., 2008), and we recently showed that DNA methylation can modulate SUR2 expression suggesting that epigenetic mechanisms may also regulate K_ATP_ subunit expression (Fatima et al., 2012). There have been no previous studies examining the effects of HDACs or HDIs on K_ATP_ subunit expression.

The results show that HDIs cause a specific increase in SUR2 expression in HL-1 cardiomyocytes but have no effect on MIN6 pancreatic β-cells indicating that HDIs have tissue specific mechanisms of action. Moreover, while HDAC-dependent histone acetylation likely contributes to the regulation of K_ATP_ expression, we made the unexpected discovery that TSA-dependent depletion of cholesterol was required for changes in SUR2 expression. Taken together the data demonstrate that HDIs specifically affect transcription of sarcolemmal K_ATP_ channel subunits and define a novel cholesterol-dependent mechanism that links HDIs with K_ATP_ subunit expression.

## Materials and methods

### Cell culture

Atrial myocyte derived HL-1 cell cultures were maintained in Claycomb medium (Sigma) (Claycomb et al., 1998). Cells were plated in tissue culture flasks coated overnight with gelatin (0.02% w/v) and fibronectin (0.5% v/v). MIN6 cells were maintained in DMEM containing 10% fetal bovine serum and 100 μg/mL penicillin/streptomycin. In all experiments to inhibit histone deacetylase (HDAC) activity, cells were incubated in standard culture medium supplemented with Trichostatin A (TSA) or Romidepsin at concentrations given in the text for 72 h. In some experiments, culture medium was also supplemented with cholesterol (250X cholesterol lipid concentrate, Gibco). Culture medium was changed daily.

### Quantitative RT-PCR

Relative expression of K_ATP_ channel subunit mRNA was examined using quantitative RT-PCR, as described previously (Fatima et al., 2012). Total RNA was isolated using a silica-based column protocol (Qiagen, RNeasy) following the manufacturer's protocols. Isolated RNA was then treated with DNAseI to digest residual genomic DNA and repurified using a silica-based column protocol. RNA concentration was determined spectrophotometrically (Nanodrop Technologies, Inc). cDNA was synthesized from 1 μg RNA (Superscript III, Invitrogen). PCR was carried out using a CFX384 Real Time PCR Detection System (Bio-Rad, Inc.), using Taqman^©^ probe and primer pairs (Applied Biosystems, Inc.) for monitoring reaction progress. Twenty nano gram of template cDNA was used in all reactions. Reactions with each primer/probe pair and template were performed in triplicate. Relative mRNA expression (compared to *Hprt*) is reported as 2^−ΔCt *^ 1000 for untreated HL-1 and MIN6 cells. The fold change in expression in response to drug treatment is reported as 2^−ΔΔCt^, where ΔΔC_t_ = ΔC_t (drug)_−ΔC_t (notreatment)_.

### Protein isolation and western analysis

Cells were washed with PBS and disrupted with RIPA lysis buffer (Santa Cruz) supplemented with protease inhibitors (Roche). Protein concentration was determined by the BCA method (Pierce). For Western analysis, proteins (typically 50 μg) were separated by PAGE and transferred to PVDF membrane. Signal was visualized with the SuperSignal West Dura ECL substrate (Pierce).

### Chromatin immunoprecipitation (ChIP)

Chromatin immunoprecipitation (ChIP) assay was performed using the SimpleChip enzymatic chromatin IP kit (Cell Signaling Technology) according to manufacturer's protocol. Briefly, 1 × 10^8^ cells were incubated with 1% formaldehyde in 1X PBS for 10 min at room temperature followed by quenching with 0.125 M glycine for 5 min. After cell lysis, cross-linked chromatin was digested with micrococcal nuclease, followed by sonication to yield fragments of 200–1000 bp. Soluble chromatin fragments were incubated with antibodies against AcH3 (Abcam, ab-10812), control IgG (Cell Signaling Technology, 2729) and H3 (Cell Signaling Technology, 4620). Bound chromatin was precipitated using MagnaChip protein A+G magnetic beads (Millipore). Precipitated complexes were eluted, decrosslinked and purified. Purified DNA was amplified by qPCR with primer set corresponding to the promoter regions (−1 kB) for SUR1 and SUR2 (Qaigen). The data was analyzed according to comparative Ct method, normalized to IgG control and reported as fold change in binding relative to control.

### Genome-wide expression analysis

MouseRef-8 v2.0 Expression BeadChips (Illumina Inc., San Diego, CA, USA) were used to measure relative levels of mRNA expression for 24,854 well-established annotated coding transcripts. Preparation of labeled cDNA, hybridization, imaging, and data collection were carried out at the Cleveland Clinic Genome Core Facility. Background subtracted, quantile normalized data were generated and analyzed using GenomeStudio (Illumina Inc., San Diego, CA, USA), GenePattern 2.0 (Broad Institute, Cambridge, MA, USA) and GATHER (Chang and Nevins, 2006) software packages. Four paired samples (treated and control) of each group were tested. Cells were treated with either TSA (30 mg/mL) or Romidepsin (5 nM). Differentially expressed transcripts were filtered using strict criteria for, accurate detection in at least three out of four samples, transcripts with 4-fold expression level differences and were regulated by both TSA and Romidepsin.

### Cholesterol labeling with filipin

The protocols were adapted from the protocols of Leventhal et al. (2001). After treatment for 72 h with TSA or TSA combined with cholesterol, HL-1 and MIN6 cells were fixed in formalin for 10 min at room temperature. Fixed cells were labeled with filipin (0.05 mg/mL in PBS, Sigma) and PicoGreen (1:1000, Molecular Probes) for 1 h at room temperature to label non-esterified cholesterol and DNA, respectively. Images were obtained using a Leica AF6000 using the same exposure between samples within an experiment.

## Results

### HDIs increase SUR2 subunit expression in HL-1 cardiomyocytes but not MIN6 pancreatic β-cells

**Figure 1:**
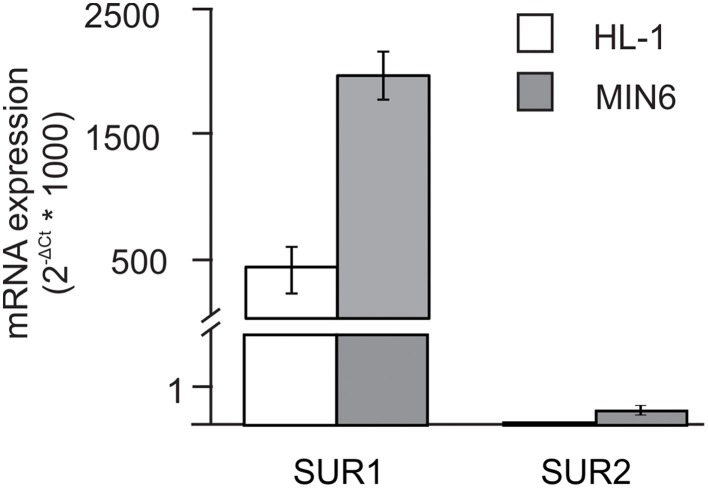
**SUR1 and SUR2 expression in HL-1 and MIN6 cell lines**. Relative mRNA expression of SUR1 and SUR2 obtained in atrial myocyte-derived HL-1 (*n* = 13) and pancreatic β-cell-derived MIN6 cells (*n* = 5). Expression (normalized to *Hprt*) was assessed by quantitative RT-PCR. Gene-specific Taqman primer and probes were obtained from Applied Biosystems. SUR1 expression was markedly higher in both cell lines when compared to SUR2.

The objective of the present study was to test the hypothesis that histone deacetylase inhibitors (HDIs) cause changes in K_ATP_ channel subunit expression. For these studies, we made use of two cell lines that express K_ATP_ channel subunits—HL-1 atrial cardiomyocytes and MIN6 pancreatic β-cells. Figure 1 shows the distribution of SUR1 and SUR2 in HL-1 cells. Expression of SUR 1 is greater than SUR2 in both HL-1 and MIN6 cells, consistent with the distribution of subunits in primary tissue (Inagaki et al., 1995b, 1996; Flagg et al., 2008). In this study, we compared the responses of these two cell types to determine 1) if HDIs modulate K_ATP_ expression and 2) if the effects are tissue specific.

**Figure 2:**
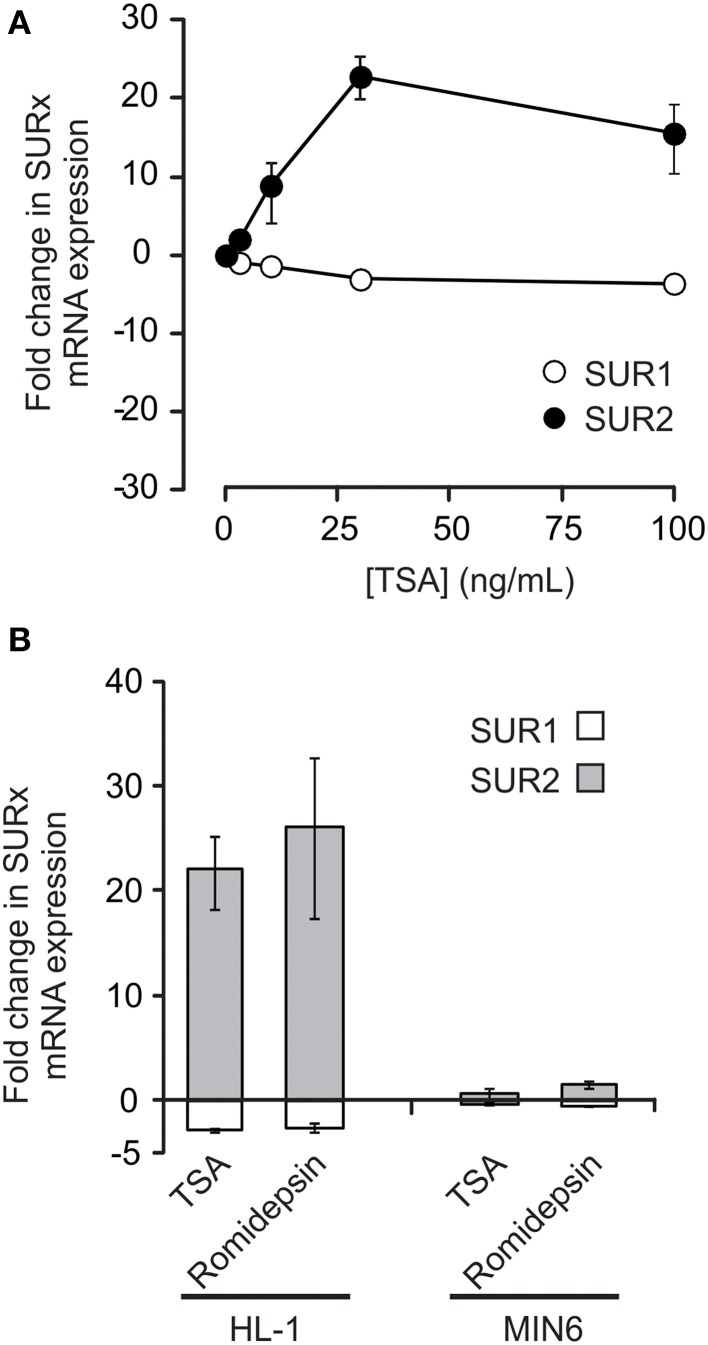
**HDAC inhibitors (HDIs) significantly increase SUR2 expression in HL-1 cells but not MIN6 cells**. **(A)** HDAC inhibition with trichostatin A (TSA) caused a marked, dose-dependent, increase in SUR2 expression, while SUR1 expression is essentially unchanged (*n* = 3–10). **(B)** Like TSA (30 ng/mL), another HDI, romidepsin (5 nM), caused an increase in SUR2 expression in HL-1 cardiomyocytes with little change in SUR1. In contrast, neither TSA nor romidepsin affected SUR subunit expression in MIN6 pancreatic β-cells (*n* = 5).

Figure 2 shows the effects of HDAC inhibition on SUR1 and SUR2 mRNA expression in HL-1 cardiomyocytes. Treatment for 72 h with trichostatin A (TSA) induced a concentration-dependent increase in SUR2 mRNA expression with little or no change in SUR1. In contrast to the results with HL-1 cells, neither SUR1 nor SUR2 mRNA expression was markedly altered by HDAC inhibition in MIN6 pancreatic β-cells (Figure 2B). Taken together, these data demonstrate that HDIs do modulate K_ATP_ subunit expression and that this occurs in a cell or tissue specific manner.

### Expression of SUR2 correlates with histone acetylation at the promoter

**Figure 3:**
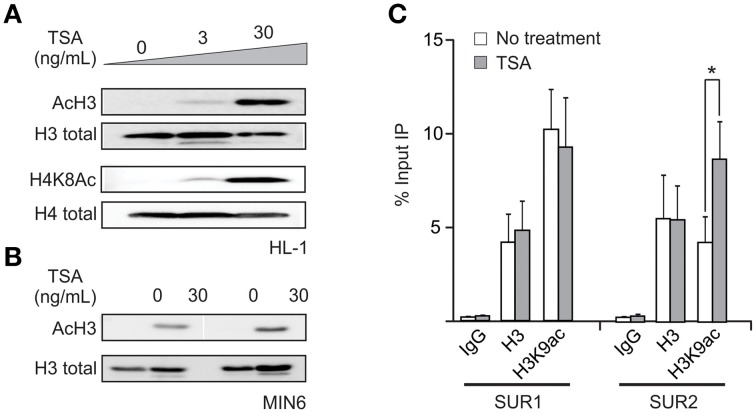
**Changes in SUR2 expression correlate with increased histone acetylation at the promoter. (A)** Representative western blot demonstrating the concentration-dependent increase in histone acetylation induced by TSA (30 ng/mL). A significant increase in both H3K9ac and H4K8ac activating histone marks, with no change in total histone protein, was observed. The concentration dependence of the TSA-dependent increase in SUR2 expression appears to correlate with the increase in histone acetylation. **(B)** Representative western blot showing that exposure of MIN6 cells to TSA causes a similar increase in histone acetylation. **(C)** Summary data from chromatin immunoprecipitation (ChIP) experiments demonstrating that the activating histone mark H3K9ac is enriched at the SUR2, but not SUR1, promoter following TSA treatment. ^*^p < 0.05.

To begin to elucidate the mechanism underlying the TSA-dependent increase in SUR2 expression, we confirmed that the HDAC inhibitors induced a global increase in histone acetylation in HL-1 cells. As expected, treatment with TSA significantly increased the amount of acetylated histone H3 and H4 without changes in the total histone protein (Figure 3A). Moreover, the increase in histone acetylation is concentration dependent and mirrors the concentration dependence of changes in SUR2 expression, suggesting that HDIs are influencing SUR2 expression through epigenetic histone modification. TSA also induces a similar increase in histone acetylation in MIN6 cells (Figure 3B). We examined the effects of TSA on gene specific histone acetylation using chromatin immunoprecipitation (ChIP). These results (Figure 3C) demonstrate a significant enrichment of the H3K9Ac histone mark at the SUR2, but not SUR1, promoter. Taken together, these data indicate that HDAC activity at the SUR2 promoter normally suppresses SUR2 transcription in HL-1 cardiomyocytes and that inhibition of this activity causes a specific increase in SUR2 expression.

### Genome-wide expression analysis reveals potential role for cholesterol homeostasis

**Figure 4:**
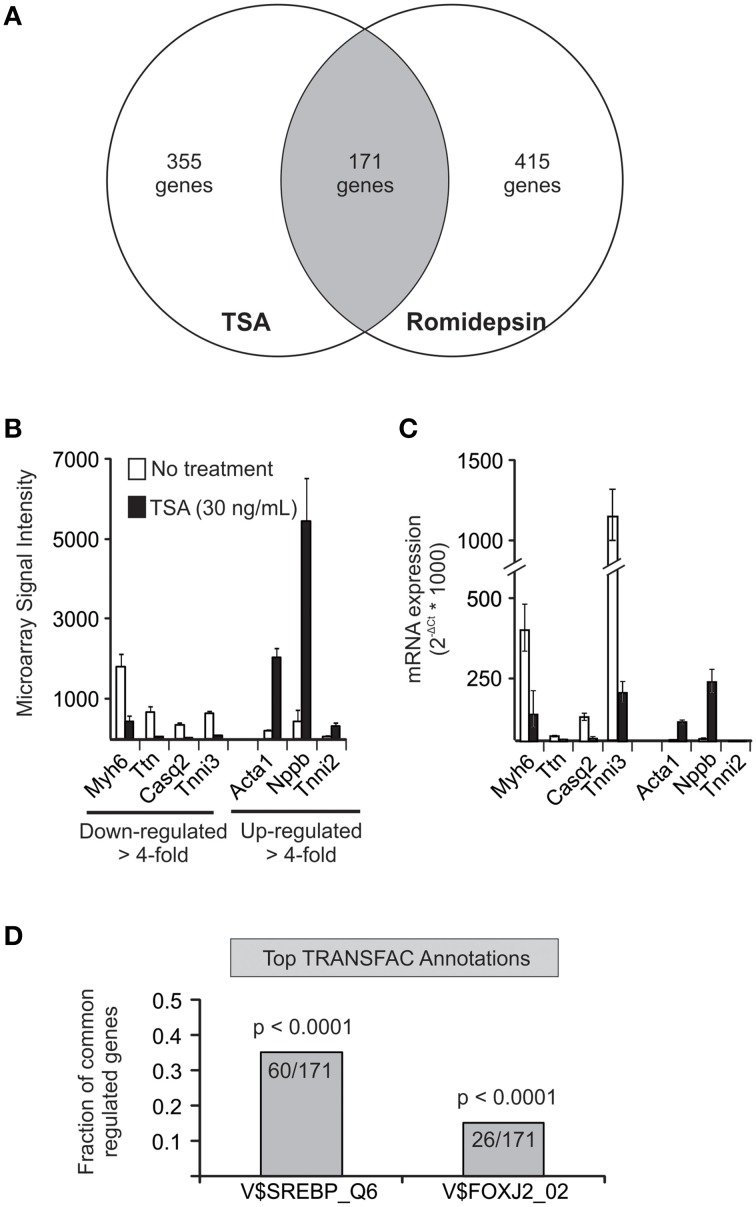
**BeadArray profiling reveals a potential role for cholesterol and SREBP. (A)** Venn diagram illustrating the number of genes specifically changed (≥4-fold increase or decrease). Analysis using GATHER software (Chang and Nevins, 2006) focused on the common gene set that was altered by both compounds. **(B)** Shown are the BeadArray signal intensities of seven transcripts that are included in the top gene ontology hit (GO0006936: muscle contraction). **(C)** Expression changes were confirmed in 5 of 7 genes tested by qRT-PCR. **(D)** Annotation of common gene set for transcription factor binding sites (TRANSFAC, v8.2) revealed a putative role for SREBP in 60 out of 171 genes annotated.

To gain further mechanistic insight, we next performed a comprehensive genome-wide expression BeadArray profiling to determine the transcripts that are up- and down-regulated by HDIs in HL-1 cells (Figure 4). Three hundred fifty five genes were found to be increased or decreased more than 4-fold in the presence of TSA. Similarly, 415 genes were changed more than 4-fold by romidepsin with 171 genes common to the two data sets. Analysis of the data set using the GATHER software (Chang and Nevins, 2006) revealed marked changes in genes associated with muscle contraction (GO: 0006936), including Myh6, Ttn, Casq2, Tnni3, Acta1, Nppb, and Tnni2. qRT-PCR analysis confirmed at least 4-fold differential expression in five of the seven transcripts that we tested (Figure 4C). One of these transcripts (*Tnni2*) was expressed at very low levels and the other (*Ttn*) was only decreeased 2.8-fold. We also annotated the 171 common gene dataset for enrichment of transcription factor binding sites (TRANSFAC v 8.2). Interestingly, this analysis demonstrated significant enrichment for sterol response element binding proteins (SREBPs) activity in response to HDIs. (60 of 171 transcripts, *p* < 0.0001). The enrichment of SREBP binding sites in the subset of transcripts affected by HDIs suggests that SREBP may play a role in mediating the effects of HDIs on K_ATP_ channel subunit expression. This possibility was wholly unexpected, however, it should be pointed out that a previous study noted the presence of SREBP binding sites in the promoter regions of the SUR2 and Kir6.2 genes (Philip-Couderc et al., 2008), and therefore we followed up this lead.

### HDIs specifically deplete HL-1 cells of cholesterol

**Figure 5:**
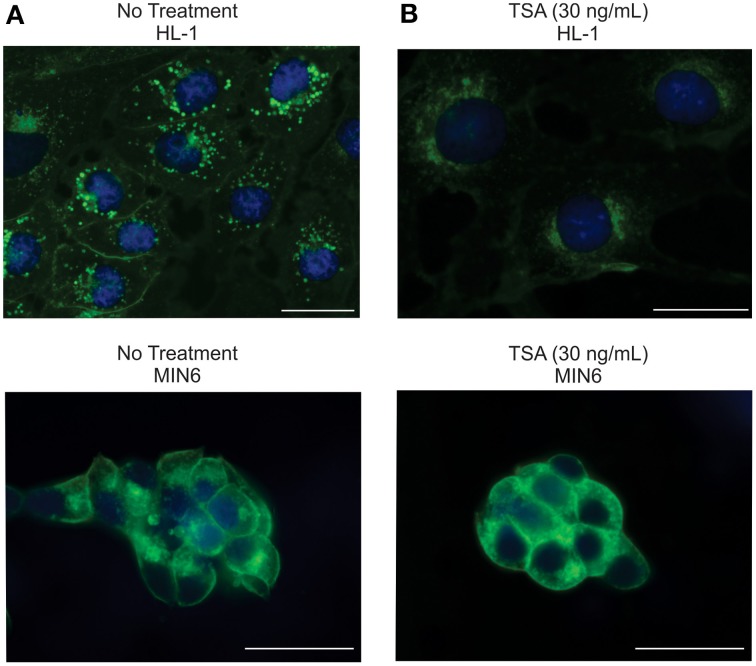
**TSA depletes cholesterol in HL-1 cells, but not MIN6 cells. (A)** Representative images of HL-1 or MIN6 cells labeled with filipin (Cholesterol, blue) and picoGreen (Nuclei, green). **(B)** Treatment with TSA (30 ng/mL) markedly reduces filipin staining in HL-1 cells but has little effect on filipin staining in MIN6 cells. Scale bar = 25 μm.

A major physiological regulator of SREBP activity is cellular cholesterol. Reduced cholesterol levels in the endoplasmic reticulum trigger a sequence of events leading to cleavage of full length SREBP, allowing translocation of the transcriptionally active SREBP cleavage product to the nucleus (Jeon and Osborne, 2012). Interestingly, recent evidence suggests that HDIs can interfere with cholesterol homeostasis (Meaney, 2014). Therefore, we investigated the effects of TSA treatment on cellular cholesterol in HL-1 and MIN6 cells cells using filipin, a fluorescent antibiotic that has been used as a qualitative measure of cellular cholesterol (Bittman and Fischkoff, 1972). TSA exposure markedly decreased filipin staining in HL-1 cells and cholesterol but did not affect filipin staining in MIN6 cells (Figure 5) suggesting that TSA-dependent cholesterol depletion might be required for TSA effects on gene expression.

### Cholesterol suppresses the effects of HDIs on K_ATP_ subunit expression

**Figure 6:**
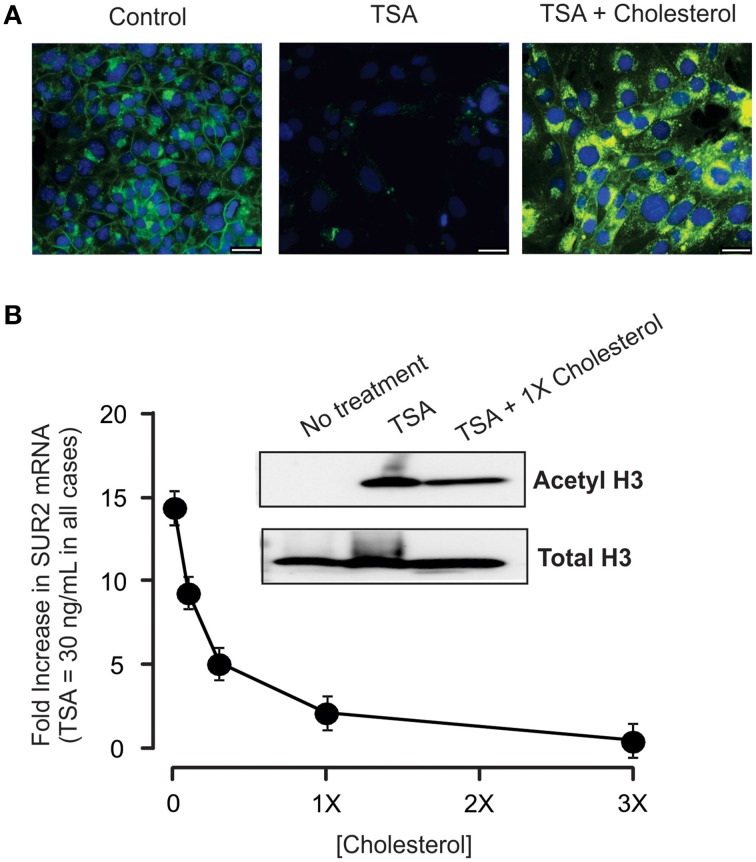
**Cholesterol abolishes the effect of TSA. (A)** Representative images of HL-1 cells treated with TSA alone or TSA + 1X cholesterol. Inclusion of cholesterol in the media prevents the marked reduction in cellular cholesterol caused by TSA. Scale bar = 25 μm. **(B)** Summary data from experiments (*n* = 5) in which HL-1 cells were treated with a constant concentration of TSA (30 ng/mL) together with increasing amounts of cholesterol. Importantly, cholesterol did not prevent the increase in histone acetylation (inset) indicating that cholesterol did not interfere with TSA access to the cell or enzymatic activity.

To determine whether TSA-dependent disruption of cholesterol homeostasis mediates the effects on K_ATP_ expression, we assessed SUR2 expression in HL-1 cells incubated in culture medium supplemented with increasing concentrations of cholesterol and a constant concentration of TSA. Addition of cholesterol to the media completely restored cellular cholesterol as evidenced by the restoration of filipin staining (Figure 6A). Cholesterol also abolished the effects of TSA on gene expression in a concentration-dependent manner (Figure 6B). Importantly, TSA continued to inhibit HDAC activity as evidenced by the increase in acetylated H3 in the presence or absence of cholesterol. Taken together, this data indicates that cholesterol depletion is required for HDI-dependent effects on K_ATP_ subunit expression in HL-1 cardiomyocytes.

## Discussion

The data in the present study demonstrate two novel aspects regarding the transcriptional control of K_ATP_ channel subunits. First, we show that inhibiting HDAC activity specifically increases SUR2 gene transcription in HL-1 cardiomyocytes. This result implies that under normal circumstances HDACs are recruited to the SUR2 gene locus in HL-1 cells contributing to the suppression of SUR2 expression in these cells under basal conditions. Second, we show that disruption of cellular cholesterol homeostasis is a critical mediator of HDI effects in HL-1 cells. This result implies that cholesterol homeostasis, in general, may be an important determinant of K_ATP_ expression in the cardiovascular system.

### Specific epigenetic regulation of K_ATP_ channel subunit expression by HDACs

Covalent histone modifications including acetylation, methylation, ubiquitylation, sumoylation, and phosphorylation are increasingly recognized as epigenetic regulators of when and where genes are actively expressed (Kouzarides, 2007). By promoting a more compact chromatin conformation, histone deacetylation is an important gene silencing factor. Here we show that inhibiting HDAC activity increases SUR2 gene transcription in HL-1 cardiomyocytes. This is the first demonstration that HDIs can influence K_ATP_ channel subunit expression providing evidence that K_ATP_ channel subunit expression is normally determined in part by HDAC-dependent epigenetic regulation.

Specificity is a key feature of epigenetic regulation of K_ATP_ channel expression. First, HDIs exhibit cell specific effects as evidenced by the observation that K_ATP_ subunit expression is markedly affected in HL-1 cardiomyocytes but unaffected in MIN6 cells. HL-1 cells appear to be primed to express SUR2, but tonic suppression by HDACs normally maintains a low level of SUR2 expression. In contrast, the appropriate transcriptional activators of SUR2 expression appear to be absent in MIN6 cells and in this case suppression of SUR2 gene expression by HDACs would be redundant and is therefore not present. In addition to cell specificity, epigenetic regulation of K_ATP_ channel expression is also subunit specific. HDIs specifically increase histone acetylation at the SUR2 gene but have no effect at the SUR1 locus. This demonstrates that HDACs are recruited specifically to the SUR2 locus in order to establish the pattern of K_ATP_ subunit expression observed in HL-1 cells.

### HDI therapy, epigenetics, and arrhythmia

Disruption of the appropriate K_ATP_ channel composition or function has been shown to cause arrhythmias or abnormal ECG responses to ischemia. Using transgenic models that overexpress K_ATP_ subunits, we have shown that disrupting normal K_ATP_ composition by overexpressing SUR1 is arrhythmogenic (Flagg et al., 2007), illustrating the importance of establishing the appropriate channel composition in the mouse heart (Flagg et al., 2008; Glukhov et al., 2010). Genetic or pharmacologic inhibition of K_ATP_, for example, completely abolishes the ST segment elevation associated with ischemia (Kubota et al., 1993; Li et al., 2000) and K_ATP_ channel openers such as pinacidil have been shown to induce ST segment elevation independent of ischemia (Yan and Antzelevitch, 1999). K_ATP_ is a particularly potent means of modulating cardiac action potential duration. Activation of just 1% of the total K_ATP_ channels available has been shown to shorten the APD by 50% (Nichols et al., 1991; Weiss et al., 1992; Knopp et al., 1999). Moreover, in ischemia models it has been shown that shortening of APD by only about 20% is correlated with marked ST elevation of about 5–10 mV (Kingaby et al., 1986). Based on these observations, it is possible that even very small changes in K_ATP_ could be manifest as changes in the ECG.

The onset of cardiac arrhythmias has been observed in several clinical and pre-clinical studies of HDI therapy (Berry et al., 2008). Ventricular tachycardia, torsades de pointes, and atrial fibrillation have been reported as rare cardiovascular events in early phase trials of HDIs (Sandor et al., 2002; Kelly et al., 2005; Piekarz et al., 2006; Lynch et al., 2012). The major cardiac effect associated with romidepsin therapy is asymptomatic, non-specific, ischemia-independent ST segment elevation or depression (Sandor et al., 2002; Piekarz et al., 2006; Noonan et al., 2013). Because ST segment elevation is typically associated with activation of K_ATP_ channels (Kubota et al., 1993; Li et al., 2000), the cardio-specific effects of romidepsin and TSA on K_ATP_ channel subunit expression provide a potential molecular basis for the cardiac effects associated with HDIs. Similarly, re-entrant arrhythmias can be promoted by activation of K_ATP_ (Billman, 2008). Other mechanisms such as decreased connexin 43 expression (Xu et al., 2013) or non-specific HERG blockade have been suggested to account for the cardiac effects of HDI therapy. Here we suggest that epigenetic alterations in cardiac K_ATP_ channel expression may also contribute to the electrophysiological events observed during HDI-therapy.

### HDIs, cholesterol, and K_ATP_ expression

The second major finding in this study is the discovery of a key role for cholesterol depletion in mediating the effect of HDIs on SUR2 expression. The key clue that focused attention on cholesterol was the association of SREBP with the gene changes induced by the HDIs (Figure 4). SREBPs are transcription factors that are typically associated with cellular lipid homeostasis (Jeon and Osborne, 2012). Previous studies have linked HDIs with a reduction of cholesterol. For example, Niemann-Pick C disease causes intracellular cholesterol accumulation as a result of mutations in the NPC1 or NPC2 gene product that are critical for cholesterol trafficking [Ory, 2004 and HDIs have been shown to largely correct the cholesterol accumulation phenotype both in fibroblasts cultured from human NPC patients (Pipalia et al., 2011) and neural stem cells from NPC^−−∕−−^ mice (Kim et al., 2007). Similarly, others have shown that TSA causes a significant reduction in cellular cholesterol by increasing expression of genes that traffic and export cholesterol as well as inhibiting expression of genes in the mevalonate pathway of cholesterol synthesis (Nunes et al., 2013). The specific molecular mechanisms driving cholesterol reduction in HL-1 cells in the present study remain to be determined as does the cell-specific basis for the HDI effects on cholesterol homeostasis. Nevertheless, it is clear that HDIs significantly disrupt cellular lipid homeostasis in HL-1 cardiomyocytes, but not MIN6 pancreatic β-cells.

The data indicate that maintaining cellular cholesterol prevents the effect of HDIs on K_ATP_ subunit expression. This finding suggests the very intriguing conclusion that there is a link between cellular cholesterol homeostasis and K_ATP_ expression. There have been no previous studies that directly link cholesterol homeostasis with K_ATP_ channel expression. *In silico* analysis previously identified SREBP binding sites in the SUR2 and Kir6.2 promoters (Philip-Couderc et al., 2008), but the functional relevance of those putative binding sites has not specifically been explored. Interestingly, there have been studies that indirectly link cholesterol homeostasis with K_ATP_ expression. Hypercholesterolemia, which is expected to inhibit SREBP, has been associated with impaired ischemic preconditioning (Csonka et al., 2014) which requires functional sarcolemmal K_ATP_ channels (Suzuki et al., 2002). It should also be noted that there is a precedent for lipid homeostasis and SREBP in regulating the expression of an inward rectifying potassium channel. Park and colleagues demonstrated that lipid lowering leads to an increase in GIRK1 expression through activation of SREBP-1 (Park et al., 2008). The present results suggest that cholesterol homeostasis and lipid metabolism in general may have analogous effects on K_ATP_ subunit expression with consequent arrhythmias.

## Conclusion

In summary, the results presented in this study demonstrate that HDIs such as TSA and romidepsin cause marked changes in the expression of K_ATP_ channel subunits. This defines a role for HDACs in determining which K_ATP_ channel subunits are expressed in cardiomyocytes but not pancreatic β-cells. Moreover, the data demonstrate a key role for cholesterol homeostasis in regulating this process. Taken together, these novel observations could lead to new strategies to modulate sarcolemmal K_ATP_ channel structure and function in the heart where it protects against stress and ischemia (Flagg et al., 2010). Moreover, our observations may explain the ST segment morphological changes that are associated with HDI therapy in cancer patients.

### Conflict of interest statement

The authors declare that the research was conducted in the absence of any commercial or financial relationships that could be construed as a potential conflict of interest.
